# Health-Enabling Technologies to Assist Patients With Musculoskeletal Shoulder Disorders When Exercising at Home: Scoping Review

**DOI:** 10.2196/21107

**Published:** 2021-02-04

**Authors:** Lena Elgert, Bianca Steiner, Birgit Saalfeld, Michael Marschollek, Klaus-Hendrik Wolf

**Affiliations:** 1 Peter L. Reichertz Institute for Medical Informatics of TU Braunschweig and Hannover Medical School Hannover Germany; 2 Peter L. Reichertz Institute for Medical Informatics of TU Braunschweig and Hannover Medical School Braunschweig Germany

**Keywords:** shoulder, upper extremity, musculoskeletal diseases, exercises, physical therapy, telerehabilitation, technology-assisted therapy, assistive technologies, mobile phone

## Abstract

**Background:**

Health-enabling technologies (HETs) are information and communication technologies that promote individual health and well-being. An important application of HETs is telerehabilitation for patients with musculoskeletal shoulder disorders. Currently, there is no overview of HETs that assist patients with musculoskeletal shoulder disorders when exercising at home.

**Objective:**

This scoping review provides a broad overview of HETs that assist patients with musculoskeletal shoulder disorders when exercising at home. It focuses on concepts and components of HETs, exercise program strategies, development phases, and reported outcomes.

**Methods:**

The search strategy used Medical Subject Headings and text words related to the terms *upper extremity*, *exercises*, and *information and communication technologies*. The MEDLINE, Embase, IEEE Xplore, CINAHL, PEDro, and Scopus databases were searched. Two reviewers independently screened titles and abstracts and then full texts against predefined inclusion and exclusion criteria. A systematic narrative synthesis was performed. Overall, 8988 records published between 1997 and 2019 were screened. Finally, 70 articles introducing 56 HETs were included.

**Results:**

Identified HETs range from simple videoconferencing systems to mobile apps with video instructions to complex sensor-based technologies. Various *software*, *sensor hardware*, and *hardware* for output are in use. The most common *hardware* for output are PC displays (in 34 HETs). Microsoft Kinect cameras in connection with related *software* are frequently used as *sensor hardware* (in 27 HETs). The identified HETs provide direct or indirect *instruction*, *monitoring*, *correction*, *assessment*, *information*, or a *reminder to exercise*. Common parameters for exercise instructions are a patient’s *range of motion* (in 43 HETs), *starting and final position* (in 32 HETs), and *exercise intensity* (in 20 HETs). In total, 48 HETs provide visual instructions for the exercises; 29 HETs report on *telerehabilitation* aspects; 34 HETs only report on prototypes; and 15 HETs are evaluated for technical feasibility, acceptance, or usability, using different assessment instruments. Efficacy or effectiveness is demonstrated for only 8 HETs. In total, 18 articles report on patients’ evaluations. An interdisciplinary contribution to the development of technologies is found in 17 HETs.

**Conclusions:**

There are various HETs, ranging from simple videoconferencing systems to complex sensor-based technologies for telerehabilitation, that assist patients with musculoskeletal shoulder disorders when exercising at home. Most HETs are not ready for practical use. Comparability is complicated by varying prototype status, different measurement instruments, missing telerehabilitation aspects, and few efficacy studies. Consequently, choosing an HET for daily use is difficult for health care professionals and decision makers. Prototype testing, usability, and acceptance tests with the later target group under real-life conditions as well as efficacy or effectiveness studies with patient-relevant core outcomes for every promising HET are required. Furthermore, health care professionals and patients should be more involved in the product design cycle to consider relevant practical aspects.

## Introduction

### Background

Health-enabling technologies (HETs) promote individual health and well-being via sensors and communication technologies [1,2]. They are information and communication technologies, particularly for the health sector. One field of HET application is telerehabilitation, a subcategory of telehealth care and telemedicine. Telerehabilitation provides and supports rehabilitation measures at a distance and connects health care professionals and patients [3,4]. An aging population, the shortage of health care professionals, especially in rural areas, and special situations with contact restrictions such as the coronavirus pandemic show the importance of telerehabilitation [5,6] and the potential of HETs for telerehabilitation [5,7]. This also applies to HETs that assist patients with musculoskeletal shoulder disorders in their home-based exercises and exercises outside of physiotherapy. Shoulder disorders are among the most frequently reported musculoskeletal problems and lead to considerable socioeconomic costs [8,9]. To maintain or improve the success of therapy, patients with musculoskeletal shoulder disorders usually perform exercises at home to complement their rehabilitation treatment (eg, physiotherapy) [10].

Although there are a few reviews on information and communication technologies to assist exercise therapy for patients with neurological diseases [11-13], an overview of technologies for patients with musculoskeletal shoulder disorders is missing. Such an overview could show the current state of HET development, the need for development, and indications for clinical use.

### Objectives

Against this background, the overall aim of this review is to identify and analyze the concepts and components of HETs, strategies of exercise programs, development phases, and reported outcomes for HETs that assist patients with musculoskeletal shoulder disorders who exercise at home. The following research questions were addressed:

1. Overview:
  - Target group: Which groups do the HETs target?
  - Objectives: What are the reported objectives of the HETs?
2. Forms of HET assistance:
  - Instruction: How do HETs assist patients with instructions on how to perform exercises?
  - Monitoring: How do HETs monitor exercise quality and quantity?
  - Correction: How do HETs correct patients’ exercise performance?
  - Assessment: How do HETs assist patients in terms of assessment?
  - Provision of information: To what extent do HETs provide additional information beyond direct assistance during the exercises?
  - Reminder: How do HETs assist patients in terms of reminding them to exercise?
  - Visualization: What forms of exercise visualization do HETs provide?
  - Telerehabilitation: To what extent do HETs use telerehabilitation aspects?
3. Strategies used by exercise programs:
  - Structure: How are HET-assisted exercises structured in terms of therapeutic goals, number of different exercises, frequency of exercise execution, and phases of the exercise program?
  - Adaptation: How can the exercises and the exercise programs in HETs be adapted?
4. HET components:
  - Sensor hardware: What sensor hardware is used to capture (motion) data?
  - Hardware: What hardware is used as output device for patients?
  - Software: Is the software off-the-shelf or self-developed?
5. Development and evaluation:
  - Interdisciplinary development: To what extent were HETs developed in interdisciplinary cooperation?
  - System status and project phase: What is the current system status or project phase and which phases have been reported?
  - Evaluation: Which (clinical) outcomes are reported?

## Methods

### Eligibility Criteria

This scoping review was conducted following the PRISMA-ScR (Preferred Reporting Items for Systematic Reviews and Meta-Analysis extension for Scoping Reviews) [14]. Inclusion criteria were defined according to the PICO (Patient or Population, Intervention, Comparison, Outcome) framework [15]. Patients were defined as patients with musculoskeletal shoulder disorders. Intervention was described as technology-assisted exercises outside of therapy sessions, specifically technology-assisted, home-based shoulder exercises. Comparators or any specific outcomes were not specified as this scoping review aims to provide a general overview. Articles on other populations (eg, adults with neurological disorders) and articles on other interventions (home-based exercises not assisted by information and communication technologies or with movement analyses unrelated to exercising) were excluded. Robots, exoskeletons, and orthoses intervening in the exercise flow in a special way were also excluded because of the lack of comparability with other technologies. Articles on studies with and without follow-up were included, and there were no restrictions by type of setting as long as the HETs were suitable for application at patients’ homes. Peer-reviewed articles in all languages were included. Articles in languages other than English or German were classified and translated by external experts.

### Information Sources

MEDLINE (PubMed interface), Embase (OVID interface), IEEE Xplore, CINAHL (Cumulative Index to Nursing and Allied Health Literature), PEDro, and Scopus databases were searched. The year 1997 was chosen as the starting point for the search because before this, the use of information and communication technologies, assistive technologies, and HETs to assist patients with their exercises was rare. The search was conducted on July 16, 2019. To maximize the coverage of literature, the reference lists of included articles and relevant reviews identified through the search were complementarily scanned by following the *pearl growing method*.

### Search Strategy

The search strategy was developed according to the PICO framework using Medical Subject Headings (MeSH) and text words related to the terms *upper extremity*, *exercises*, and *information and communication technologies*. The specific search strategies were developed by a medical computer scientist and a physiotherapist in consultation with the review team and 2 librarians experienced in systematic literature searches. The MEDLINE strategy was adapted to the syntax and subject headings of the other databases. The search terms are included in Multimedia Appendix 1.

### Selection, Categorization, and Data Extraction

Literature search results from each database were imported into the literature management program *Citavi* (*Citavi 5, Swiss Academic Software*). Duplicates were removed by PubMed ID, Digital Object Identifier, and International Standard Book Number (ISBN).

Two reviewers (LE and B Steiner) independently screened the titles and abstracts against predefined inclusion and exclusion criteria. Full texts were obtained for all titles that met the inclusion criteria or where there were uncertainties. The 2 reviewers screened all full texts for final inclusion. Disagreements in both screening processes were resolved through discussion. Persisting disagreements were resolved through discussion with a third party from the review team (B Saalfeld or KW). The reasons for excluding articles were recorded and categorized according to the fulfilled exclusion criteria (only the first matching criterion). Overlapping or accompanying articles describing the same HET were included and specified in a summary table. Only the main article was included in the overview.

To ensure consistency between the 2 reviewers, a pilot data extraction was conducted on 5 randomly selected articles of the included full-text articles. The 2 reviewers independently extracted data from these 5 articles. Disagreements on categorization were resolved through discussion. Persisting disagreements were resolved through discussion with a third party from the review team (B Saalfeld or KW). One reviewer (LE) then extracted the data from all other eligible full-text articles based on the consensus reached during discussion of the 5 articles.

### Synthesis of Results

A systematic narrative synthesis was performed with information presented in texts and tables to explain the characteristics, categories, and findings of the included articles. A coding frame with categories and subcategories was built in a mix of concept-driven and data-driven approaches (deductively-inductively) [16]. The main categories *form of HET assistance*, *exercise program strategy*, *HET components*, *system/project phase*, and *reported outcomes* were defined as concept-driven after literature research and unstructured expert interviews. The 2 categories *interdisciplinary development* and *adaptation*, along with further subcategories, were derived from the texts using a data-driven approach in the form of a growing list. All coded categories and subcategories can be found in the Results section and in Multimedia Appendix 2. For better identification, main categories and subcategories in the text are written in italics.

## Results

### Overview

**Figure 1:**
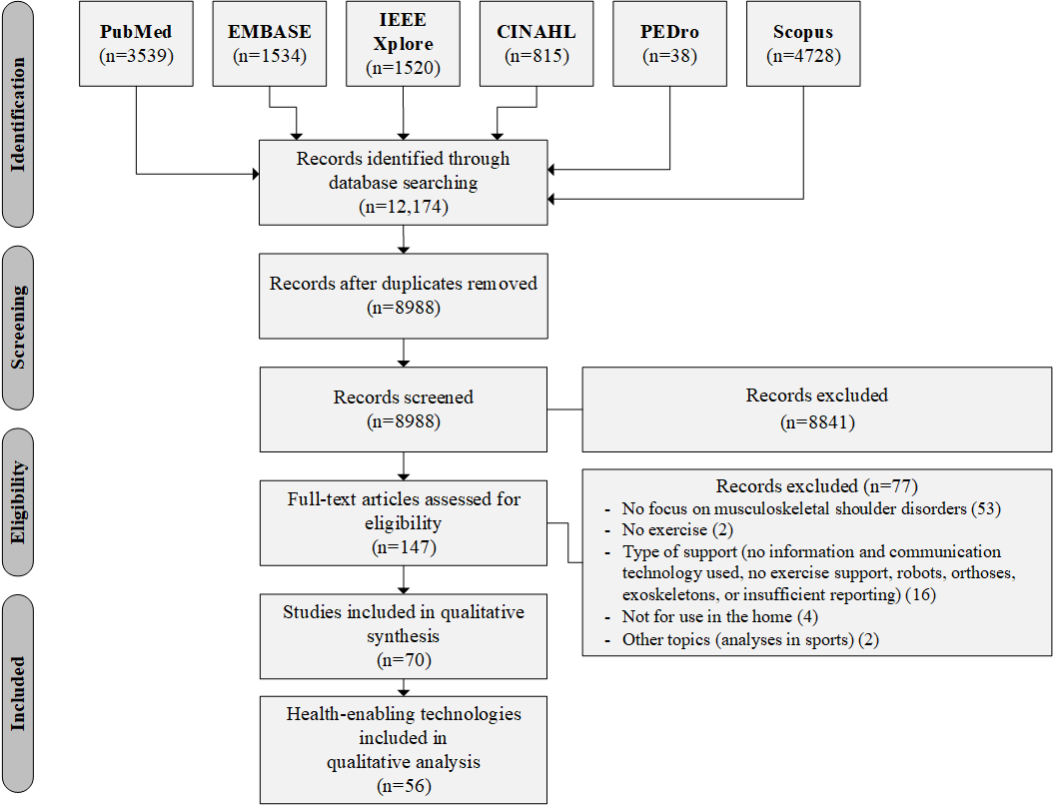
PRISMA (Preferred Reporting Items for Systematic Reviews and Meta-Analysis) flow diagram.

The *PRISMA flow diagram* in Figure 1 (adapted from [17]) provides an overview of the literature search. Multimedia Appendix 2 contains the complete table of articles and analysis categories. A total of 70 articles introducing 56 HETs were included in this review. The 70 identified articles differ according to the target group and their overall objectives.

All identified articles and related HETs, grouped by telerehabilitation aspects, are shown in a table in Multimedia Appendix 3 [18-87].

#### Target Group

All 56 articles describe HETs that can help patients with musculoskeletal shoulder disorders when exercising at home (Table 1). Of these, 18 articles refer to a specific target group for their use [20,23,26,36,37,45,47-50,52,54,58,62,68,71,73,77]. In 11 articles, the HET is recommended for several target groups [19,30,31,42,53,55,60,75,76,81,83]. Cubukcu and Yüzgec [55], for example, address patients with shoulder joint, muscle, and tendon damage. In 5 articles, the focus is on patients with musculoskeletal shoulder problems and patients with neurological disorders (eg, stroke) [42,76,80,81,83]. In total, 27 articles do not directly name the proposed target group [29,34,38,40,43,44,46,51,52,55-57,59,61,65-67,69,70,72,74,78,80,82,84-87].

**Table 1:** Target groups connected to musculoskeletal shoulder disorders in descending order of frequency.

Target group	Frequency, n (%)	References to health-enabling technologies
Frozen shoulder	18 (32)	[23,31,36,37,42,45,47-49,54,58,60,62,66,70,76,81,83]
Shoulder impingement syndrome	11 (20)	[24,31,50,55,60,68,70,71,73,75,81]
Rotator cuff tear	8 (14)	[20,31,55,60,70,75,80,81]
Humerus fracture	7 (13)	[26,31,53,55,60,70,81]
Rheumatoid arthritis	6 (11)	[31,55,60,70,77,81]
Arthrosis	5 (9)	[31,55,60,70,81]

#### Objectives

*Assistance with home exercises* and *monitoring exercises* are the most reported HET objectives (34/56, 61%). *Simple instructions* are reported for 12 HETs. Only one HET aims at patients’ *reintegration into employment* [32].

A total of 14 articles describe a specific period of use. This period ranges from 3 weeks [69] to 12 weeks [24,54] to 6 months [20]. The reason given for these periods is the underlying study design, inappropriate therapeutic follow-up time, or the duration of the rehabilitation phase.

### Forms of HET Assistance

The HETs assist patients with their exercises by instructing, monitoring, correcting, assisting with assessments, providing additional information, and reminding them about exercising (Table 2).

The *instruction*, *monitoring*, and *correction* of exercises, as well as the *provision of additional information*, is carried out directly or indirectly. Direct HETs instruct patients on specific movements, give feedback on movement performance or provide additional information (eg, how to modify daily activities [20]). Indirect assistance occurs in 2 different ways or in a combination of both. Either therapists assist directly using HETs (eg, watching videos of patients [29] and receiving accumulated data for interpretation [23]) or HETs instruct, monitor, or correct patients’ movements indirectly by playing games [31].

Other ideas for supporting patients while exercising at home are described in the discussions of the identified articles but have not yet been realized. These ideas are mentioned in Table 2 under “Implementation planned.”

**Table 2:** Assistance options in descending order of frequency.

Availability HET^a^ assistance	Available (%) (direct (%) + indirect (%))	Implementation planned, n (%)
Instruction	50 (89) (19 (34)+31 (55))	0 (0)
Monitoring	40 (71) (34 (61)+6 (11))	4 (7)
Correction	36 (64) (9 (16)+27 (48))	4 (7)
Assessment	26 (46) (26 (46)+0 (0))	4 (7)
Additional Information	7 (13) (5 (9)+2 (4))	0 (0)
Reminder	4 (7) (4 (7)+0 (0))	0 (0)

^a^HET: health-enabling technology.

#### Instruction

Usually, patients need instructions on how to perform exercises correctly. This subsection focuses on (1) whether it is specified who gives the instruction, (2) in which form and with which movement parameters, (3) the timing, and (4) which visual, auditory, or tactile types of assistance are used in the exercise programs.

In total, 47 articles report instructions given by HETs, whereas 3 articles report guidance by therapists alone [26,29,51]. In 6 articles, HETs and therapists instruct exercises together [19,23,24,43,53,59]. Pastora-Bernal et al [24], for example, provide training videos with exercise instructions, and the therapist enhances this via videoconferencing. With the *iJoint App*, the therapist guides the patient while the app provides information about target angles, actual angles, number of repetitions, and beeps when a target angle is reached [23]. A total of 25 HETs indirectly instruct exercises using games.

Both direct and indirect exercise instructions are mostly given using *range of motion* (ROM; 43/50, 86%). Other parameters related to movement execution are *starting and final position* (32/50, 64%), *smoothness of movement* (5/50, 10%), *speed* (18/50, 36%), *strength* [53], and *correct posture* [59]. Furthermore, 26 articles address a *training framework* for exercise instruction, that is, a kind of strategic planning of the exercise is described. At least one training science component of an exercise program must be named to fulfill this category. This can be the intensity of the exercises, for example, the *intensity* (20/50, 40%) and the *scope* (12/50, 24%) are most frequently mentioned. Only 2 articles report on *frequency* [23,58] or *density* [58,61] with regard to correct exercise performance.

All exercise instructions are given *synchronously*, that is, the patient is instructed before performing the exercise or while exercising. The *Shoulder Physiotherapy Application*, for example, provides visual instructions using skeletal images and text messages about correct exercise execution [55]. Two articles describe both *synchronous* and *asynchronous exercise instructions* via videos and written feedback [20,24]. The asynchronous part of the exercise instruction is done later via a supplementary paper-based document with an overview of the exercises [24] or written feedback with exercise instructions via email [20].

The type of assistance ranges from *visual* to *auditory* to *tactile* instructions for the exercises. Most articles describe visual assistance using *symbols* (n=33), *messages* or *texts* (n=25), *avatars* (n=22), *videos* (n=14), *schemes* or *models* (n=11), *skeleton images* (n=2), and *photos* (n=1) in different combinations. For example, the *Kinect-based telerehabilitation system* (*KiReS*) depicts the current and target status of movement with two 3D avatars and shows repetitions, series, next posture, and motivational messages. A 3-level color scale indicates whether a patient has reached a posture [19]. Pekyavas and Ergun [71] and Rizzo et al [73] use the Wii games of boxing and bowling with visual, auditory, and tactile exercise guidance.

#### Monitoring

Some HETs can monitor the quality and quantity of the performed exercises. Monitoring makes it possible to either give direct feedback to the exercising patient or inform the therapist about the patient’s current state or long-term development. The degree of detail in monitoring ranges from simply recording the information that training took place on a certain day [58] to indicating how many repetitions of an exercise were completed [56] to storing aggregated data on ROM and the recognition of compensatory movements [31]. In addition, 34 HETs monitor exercises directly, whereas 6 use indirect monitoring (Table 2); 2 articles report indirect monitoring solely by physiotherapists during videoconferencing [26,28]; 4 articles report on therapists who monitor exercises using HETs, and 10 report on both HETs and therapists who monitor the exercises. This is done, for example, by physicians and therapists evaluating recorded videos [20]. *Passive registration* of exercise execution means that monitoring starts automatically when HET-assisted exercising starts. This is described in 34 articles, whereas in 5 other articles, patients must activate the control (eg, recording ROM) to compare and track improvements [48]. A HET named *PARC*, for example, shows records of the scores and repetitions for the prescribed exercises. Physiotherapists can view exercise videos and results based on ROM measurement [43].

#### Correction

The category *correction of exercises* indicates that the patient’s exercise performance is corrected in some way. This category also specifies by whom corrections are given, in which form, and with which parameter feedback is given. The timing of correction and parameters concerning the correction of movements are stated in the last paragraph of this subsection. In total, 9 HETs provide direct correction of the exercises, the other 27 HETs correct the exercises indirectly, and 4 HETs plan to fulfill this function (Table 2).

There are instances in which therapists correct exercises while an HET serves as an aid, as is the case in a videoconferencing system [20,26,29,43]. Simultaneous correction by HETs and therapists also occur [23,45,46,51,65,73]. One example of how correction by an HET is implemented is the use of red and green buttons to indicate right and wrong movements. Popup messages provide additional explanations of correct movements [70].

The most common form of feedback is *visual feedback* (25/36, 69%). The articles report the following subcategories of visual feedback in descending order of frequency: *messages/text*, *symbols*, *schemes*, *video*, *avatar*, and *skeleton imaging*. *Auditory feedback* is characterized in 13 articles as either *sounds* or *verbal explanations*. Furthermore, 3 HETs provide *tactile/haptic feedback*, two of which use Wii games [71,73]. One single HET provides both visual, auditory and haptic feedback. It displays symbols that change color in a web application, gives auditory feedback (“keep on” or “sit straight”), and includes a module on a vest that vibrates to indicate incorrect posture [57].

Almost all HETs offering exercise corrections provide *synchronous correction* (33/36, 92%). Two HETs exclusively use *asynchronous correction* via written feedback [20] and changes in game settings by therapists for indirect correction of patients’ movement performance [20,43]. Parameters for the correction of movement execution are *ROM* (n=28), *starting and final position* (n=25), *speed* (n=9), and *smoothness of motion* (n=4).

#### Assessment

The category *assessment* is concerned with all kinds of assessments from movement measurements to questionnaires provided by HETs. The forms of *data collection* (*passive* or *active*), *timing*, and *content* are categorized (Multimedia Appendix 2). A total of 26 HETs provide assessment functions. All HETs perform assessments passively, usually during each exercise session. Active recording (eg, by pressing a monitor button) is also possible [44,58]. Most HETs evaluate the *ROM* (25/26, 96%). Four of the technologies enable therapists to supplement the assessment with patient-reported outcomes regarding *pain*, *strength*, or *function* [19,31,42] or to calibrate the neutral position and range of allowed movements [59]. Patient-reported outcomes are actively provided and entered by patients [19,31,42]. In Anton et al [19], therapists are also able to create individual questions.

#### Provision of Information

The category *provision of information* includes all additional information beyond direct support for exercise execution. In total, 7 articles report on this topic. Of these, 2 articles describe a given structure for *videoconferences* to do so. Structural elements include a question period [26] and a three-way meeting with patients, outpatient physiotherapists, and physiotherapists at the hospital [29]. A total of 5 HETs provide information as a *tutorial* that shows *how to use the HET* [62], *how to use the wearable devices* [37]*,* “*information on different care activities and how to modify daily activities*” [20], “*on-screen tips about the importance of exercising*” [70], and *a display screen showing “a brief definition of frozen shoulder, [...] common treatment options, pain medication, and mobilization exercises*” [58]*.*

#### Reminder to Exercise

A total of 4 HETs remind patients to exercise. One article reports a *calendar reminder* and a status report for exercises for each training session [58]. The other 3 articles do not specify the implementation of this function [23,37,54].

#### Visualization

Exercises are visualized in different ways: *2D or 3D graphics* and *aggregated information* mostly visualize guidance or exercise performance (eg, by *ROM values*, *speed in graphs*, and *real-time videos* [46]). Aggregated information can take the form of graphs or scores in a game. The subcategories *augmented reality*, *augmented virtuality*, and *virtual reality* can be thought of on a continuum between physical reality and virtual environment according to Milgram et al [88]. No subcategories for physical reality were created. The other 3 subcategories were created in a data-driven manner. To be classified as virtual reality, both the visualization of the exercises and feedback during the exercises must take place in a virtual environment. Augmented reality indicates that the virtual and physical environment are mixed. If, in this mix, a Red Green Blue (RGB) image is visualized in a virtual environment, then it is classified as augmented virtuality and, as such, a subcategory of augmented reality. In total, 15 HETs use *virtual reality*, 15 use *augmented reality*, and 5 use *augmented virtuality*. Sveistrup et al [83], for example, show an RGB image of the patient in front of a soccer net in a virtual soccer environment where the patient has to stop balls from scoring.

#### Telerehabilitation

The category *telerehabilitation* deals with the rehabilitation measure of exercise assistance at a distance. The connection between patients and therapists and the communication between them with the help of HETs is considered in terms of the aim of *communication*, *initiation of contacts*, *timing and communication channel*, and *content of messages*. Message content is subcategorized in *movement execution*, *framework for training*, *display of training*, *assessment*, and *aggregated information*.

Table 3 gives an overview of HETs using (20/56, 36%) or planning to use (7/56, 13%) telerehabilitation with mobile apps and game components or one of the two to assist patients in performing their exercises.

**Table 3:** Health-enabling technologies with telerehabilitation combined with apps and game components.

Number of subject	HET^a^, n (%)	HET using apps, n (%)	HET using game components, n (%)
Telerehabilitation	20 (36)	18 (32)	14 (25)
Telerehabilitation planned for the future	7 (13)	3 (5)	3 (5)
No telerehabilitation	29 (52)	17 (30)	17 (30)

^a^HET: health-enabling technology.

Telerehabilitation contacts are usually initiated by therapists to check exercise results. For example, therapists log on to a therapist portal to view patients’ exercise parameters in graphs and videos [46]. In total, 18 HETs use *web interfaces* as a communication channel. Additional communication channels include *video chats* [26,30], *video messages* [20,46], *text messages* [50], and *emails* including video recordings of exercises customized for a patient, images, and parameters of each exercise [24]. Eriksson et al [29] report on a classic *videoconference*−*based* telerehabilitation. The timing of telerehabilitation contact is mostly not stated. In total, 5 articles report *periodic* telerehabilitation meetings (eg, twice a week [20]). The *MoMo* app provides telerehabilitation contacts *on demand* [37].

The *content of messages* is largely consistent with the categories and subcategories described above for instruction, monitoring, and correction. Information on *ROM* (n=11), *starting and final position* (n=10), *speed* (n=8), and *smoothness of motion* (n=5), as well as *assessment results* (n=10), *aggregated information* (n=9), *videos* (n=7), *avatar images* (n=4), *patient images* (n=4), and *photos* (n=1) are displayed. *Aggregated information* concerns *execution of exercises* (n=8), *exercise frequency* (n=7), *number of repetitions* (n=7), and *execution quality* (n=5). This can take the form of a patient’s avatar movements from different rounds, target angle, arm side, date, time, number of repetitions, and ROM results in graphs [36]. *Intensity* (n=9), *scope* (n=9), and *frequency* (n=4) represent the *exercise program framework*.

### Strategies of Exercise Programs

#### Structure

This section describes HET-assisted exercises and exercise programs. Typical therapeutic goals of exercising for patients with shoulder disorders are reported. The most common goal is to *maintain or improve shoulder mobility* (30/56, 54%). This is followed by *strengthening* (14/56, 25%) and *pain relief* (13/56, 23%). Less frequently reported goals of technology-assisted exercises are *initiation of scapulothoracic rhythm* [24,31,57,73], *humeral head centering* [24,31,73], *postural control* [26,37,57], *increasing blood circulation* within the affected area for faster recovery [53], *motor learning* [82], and *increasing functional ability and occupational performance* [77]. In total, 23 articles did not specify any goal for the implemented exercises.

Depending on the intended use and therapeutic goal, an exercise program can be designed differently in terms of the number of exercises, frequency of exercise, and exercise duration. The category *number of assisted exercises* represents the number of different supported exercises. This number is given for 18 HETs and ranges from 2 [31] to 9 [42]. Carbonaro et al [31], for example, describe 2 exercises to externally rotate the shoulder and abduct to 80° with an elastic band. Inertial measurement units identify compensatory movements during these exercises. Rahman et al [42] defined 9 different exercises (eg, shoulder flexion and shoulder extension) for mobilization with starting and final position and integrated them into the game *Pluck the Fruits*. An app instructs wiping movements for shoulder mobilization in patients with a frozen shoulder along with 3 other exercises in Stütz et al [58]. The counting of the exercises in this category follows the authors’ definition of the exercises.

*Duration per exercise (program) performance* ranges from 5 [54] to 60 min [77]. The *exercise frequency per week* ranges from twice a week [47] to 14 times a week [26] with a recommended exercise frequency of once [58] to 3 times per day [54]. As justification for these recommendations, almost all articles mention aspects of study design. Only Chiensriwimol et al [36] explain that the treatment of a frozen shoulder requires an exercise duration of 12 to 18 months with daily exercises.

Training therapeutic exercise programs for mobilization and strengthening can be roughly divided into *warm-up phase*, *main phase*, and *cool-down phase*. Only 5 of the 56 articles report on an exercise program with a *warm-up phase* and *main phase* [26,71,73,77,83]. Two of them also mention a *cool-down phase* [71,73], and one refers to a phase in which patients can ask therapists questions [26].

#### Adaptation

*Adaptation of exercises or exercise programs* describes the possibility of adapting exercises or exercise programs to fit patient-specific characteristics, needs, or training progress. This is possible in 36 HETs. The most common criterion for adaptation is the *ROM* (28/36, 78%). In total, 24 HETs adjust the settings directly to the patient’s ROM, and 4 articles report on therapists using ROM to adapt to exercises. Other criteria are the *individual patient* (20/36, 56%), *exercise duration* (4/36, 11%), *age and gender* [34], *patient’s proportions* [55], *patient’s disease* [19], and *patient’s home environment* [29]. However, adjustment to patients is usually not described in detail. For example, Du et al [66] report adjusting the game settings and difficulty levels to fit each patient’s condition and demands without explaining how this is done. In total, 14 articles report on the adaptation of exercises during the course of therapy, and 22 articles report on the adaptation of exercises at the beginning of therapy.

Most often, therapists decide on the adjustment (28/36, 78%). Seldom do HETs adjust exercises independently (eg, adapt game levels according to a patient’s ROM [37]). Good interaction between the therapist and HET is visible in *KiReS* and *iJoint App*. *KiReS* supports therapists’ exercise decisions by assessing the rehabilitation phase based on the *TrhOnt* ontology [19]. The *iJoint App* calibrates settings via ROM, whereas physiotherapists undertake adjustments to fit a patient’s progress [36]. In total, 2 HETs allow patients to make additional adjustments and choose levels of difficulty [26,46].

### HET Components

Various HET components, such as *sensor hardware*, *hardware for output*, and *software*, are used to assist patients in their exercises. A total of 47 HETs are *transportable*, 9 are *body wearable*, and 6 are transportable technologies with wearable components. Fixed installed HETs are not among the identified HETs. One reason for this is that only HETs suitable for use in patients’ homes are included.

#### Sensor Hardware

The depth-image camera Kinect from Microsoft is the most frequently used sensor hardware. In total, 27 articles report on HETs based on *depth-image cameras* and all of them use the Kinect. For 23 HETs, the version is not specified, and one uses *Kinect for Xbox 360* (Kinect v1) [19] and 3 use the newer version *Kinect for Windows* (Kinect v2) [38,51,69]. *Inertial Measurement Units* (IMU) are part of 15 HETs, and 14 HETs use *accelerometers*, 12 *gyroscopes*, and 10 *magnetometers*. Some HETs use multiple sensors. For example, Yeh et al [45] combined joint angle measurements from IMU and Kinect v1 in their HET *cloud motion-sensing rehabilitation system*. In addition, 7 articles describe the use of sensors in smartphones. In three of them, an accelerometer, a gyroscope, and a magnetometer are used [23,36,58]. Smartphone cameras are also considered sensors in smartphones. A total of 4 HETs use the *smartphone camera* and 5 HETs have a *conventional color camera*; 2 articles report on the *Wii Nunchuck Controller* and on the *Wii Remote* [71,73]. In addition, 7 other *controllers* are used including other gaming controllers [30,62,77], a mouse [26], a red glove for a virtual reality system [83], a force feedback device [82], and a standard shoulder wheel [33,34]. The shoulder wheel has a control module for converting wheel rotation into control signals for 6 exergames. In one of the games, for example, arrows are fired at a target with the shoulder wheel at the correct angle [33,34].

#### Hardware for Output

In total, 34 HETs use *PC displays*, 11 use *smartphones*, and 8 use *televisions* as hardware for output; 6 *bigger screens* (>40) or *projectors* [26,34,37,40,47,67] and 3 *head-mounted displays* [26,46,60] are also reported. Furthermore, 6 articles do not specify the hardware [38,49,66,69,72,75]; however, these 6 articles describe interfaces for games that require visual control by the patient.

In addition, 3 *haptic devices* [57,60,82], 2 *audio-biofeedback modules* [23,35], and 3 *LEDs* controlled by an analog-digital converter with a *microcontroller* [53] are used for output.

For most technologies, the output channel is *visual*. In total, 22 articles report on *auditory* and 5 on *haptic* channels [57,60,71,73,82]. Underreporting of auditory output channels is possible because not all articles on games indicate their likely use of auditory channels. For example, Powell and Powell [72] describe the sound of falling fruit for the game of fruit picking, whereas Rahman et al [42] do not mention this for a similar game.

#### Software

In total, 9 HETs use *off-the-shelf software* [26,29,44,71,73,75,77,80,83], whereas the basis for all other technologies is *self-developed software*. Two technologies use both off-the-shelf and self-developed software [26,44]. One article about a telerehabilitation platform does not specify the software used or developed [20]. Software development is described in varying detail. This ranges from a detailed description of each developmental step [85] to a simple presentation of the programming language and game engine [38] to no description at all [57].

### Development and Evaluation

#### Interdisciplinary Development

An interdisciplinary contribution to the development of HET is mentioned in 17 articles. This includes the *development* of the technology by computer scientists or engineers and therapists or physicians, *consultation* of therapists or physicians during the development, or at least the involvement of therapists or physicians in the *evaluation*. Patients evaluated 18 HETs. In 2 articles, patients were involved in the development above and beyond this evaluation [37,74]. Chung and Chen [37] conducted a 2-month observation of the therapy process and interviewed therapists, physicians, and patients. Shi and Peng [74] performed a user requirements analysis with patients using the *house of quality method*.

In 7 other articles, an interdisciplinary development can be assumed but is not described explicitly [24,45,47,49,54,56,72]. For example, the analysis of therapeutic goals and actions is stated, but an interaction and collaboration with health care professionals and patients is not described [72].

Compared with other articles, the 17 that had interdisciplinary cooperation show an above average proportion of *provision of information* by HETs (4/5), *reminder to exercise* by HETs (4/4), *adaptation of exercises* to an individual patient (4/5), and *correction* under the telerehabilitation aspect (6/7). A relatively small proportion of these are seen in the corrections by HETs (3/9) and among the articles that do not specify a goal for the presented exercises (5/24).

#### System and Project Phase

The category *system or project phase* is based on the study phases of trials for drugs and medical devices. The included articles report tests in *phases 0, 1, 2, and 3*. None of the studies on long-term effects dealt with *phase 4*. In the following summary, an HET can be counted in multiple phases. Whenever it is reported in the corresponding articles, the already completed phases and the current project phase are recorded. Da Gama et al [65], for example, report *phase 0* and *phase 2*.

*Phase 0* is concerned with the testing of prototypes and prototypical tests; 48 articles are in *phase 0*, 34 of them end in *phase 0*, 13 articles present *initial prototypes*, 33 present *system prototypes* in which the later function is fully implemented, and 24 articles test the HET prototype under *laboratory conditions for feasibility, acceptance, usability, or safety.*

In *phase 1*, an HET is tested for *feasibility*, *acceptance*, *usability*, or *safety* in the setting (a patient’s home or a rehabilitation facility) or under everyday conditions. In total, 15 articles are concerned with *phase 1*. In 7 articles, study staff supported the tests, whereas the other 8 articles did not provide personal support. *Phase 1* is the last phase to be reported in 7 articles.

**Figure 2:**
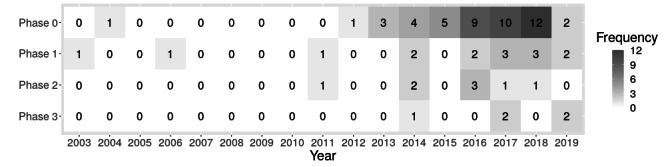
Overview of current and completed system and project phases per year.

*Phase 2* involves proof of concept and the exclusion of risks and side effects. A *first effectiveness or efficacy study* is also possible in *phase 2*. The first phase 2 testing of an HET occurred in 2011. An initially suspected increase in the later study phases over time was not found (Figure 2). *Phase 2* is reported in 8 articles, where it is also the last phase; 4 articles report on the *proof of concept*, 6 on a *first effectiveness or efficacy study*, and 1 on the *exclusion of risks and side effects* [73].

*Significant effectiveness* or efficacy is evaluated in *phase 3*. This is the case in 5 articles. For outcomes, see the section *Evaluation*. Figure 2 shows an overview of the current and completed system and project phases per year. The data for 2019 may be biased because articles were only included until July 2019.

#### Evaluation

*Feasibility*, *usability*, *acceptance*, or *effectiveness or efficacy* were tested in 49 HETs. This evaluation ranges from self-designed interviews and questionnaires [20] to the use of validated survey instruments (eg, *System Usability Scale* in [58]). Four articles report the absence of *(serious) adverse events* [26,54,73,77]. The other articles do not mention *adverse events*.

*Feasibility tests* of partial components or individual algorithms are reported (22/49, 45%), as well as tests of the entire technology (30/49, 61%). Only Pastora-Bernal et al [24] and Eriksson et al [29] describe embedding in the care process under everyday conditions.

*Usability tests* were conducted for 15 technologies. *Healthy volunteers* [20,42,48,57,70], *patients* [19,29,48,49,54,58,65,69,74], and in a few cases *therapists* [36,59,65] were interviewed or filled out a questionnaire. Mostly, relatively small samples of 3 to a maximum of 20 patients and 5 to a maximum of 11 therapists were queried. Only Choi et al [54] tested usability with the *Usefulness, Satisfaction, and Ease of Use* questionnaire in 42 patients. Two articles describe preliminary usability results with 1 patient [30] or tested only an interface prototype [37]

*Acceptance* is examined for 14 HETs in patients and 3 HETs in therapists [51,59] or physicians [42]. The level of detail in the description of user groups and sample size varies widely. For example, acceptance is tested in 1 physician [42], 12 therapists [51], 50 patients [81], or 100 users with and without impairments [72].

In total, 8 articles show significant improvements after training with assistance from the respective HET in one or more of the following categories: *mobility/flexibility* [20,29,47,49,65,73,77], *pain* [20,49,71,73,77], *strength* [20,77], *quality of life* [29,73], *activity performance* [49,71,77], *participation* [77], and *postural control* [57]. The study designs differ considerably, as do most survey instruments. Only ROM measurements were analyzed in 7 of the 8 articles. Eriksson et al [29] report a significant improvement in ROM and health-related quality of life in a nonrandomized controlled trial with 10 patients in the intervention group and 12 patients in the control group over 2 months. In their noncontrolled study in 11 patients over 6 months, Macias-Hernandez et al [20] show a significant improvement in pain on the *Visual Analogue Scale* and in muscle strength and function with the *Constant Murley Score*.

## Discussion

### Principal Findings

#### Overview

The *target group* is mainly described as typical patients with musculoskeletal shoulder disorders. It is surprising that about half of the articles offering exercise assistance do not specify their target group. A total of 5 HETs assist both neurological patients and patients with musculoskeletal shoulder problems. This is conceivable as long as the exercise goals are identical (eg, to improve mobility); however, it should be noted that the need for assistance and support can vary considerably.

Most HETs have been developed for single parts of the therapy process involving exercises, as can be seen from the reported objectives. Only Pastora-Bernal et al [24] and Eriksson et al [29] describe embedding them in the care process. Beyond this, Anton et al [19] already provide support in the selection of exercises and recommended the use of their HET, *KiReS,* in addition to regular therapy sessions.

The study design is stated as the reason for the very different periods of use. Substantive reasoning that includes the course of healing, guidelines, or expected rehabilitation phases is missing.

Some HETs offer patients a complete and balanced exercise program that follows scientific training aspects, although the program is usually not individualized. Most technologies, however, fall far short of this and cover, at most, single components and goals, such as maintaining shoulder mobility in a specific direction of movement.

#### Forms of HET Assistance

The concepts underlying the assistance provided by HETs are subcategorized into *instruction*, *monitoring*, and *correction* with the subsubcategories *direct and indirect instruction, monitoring, and correction*. In physiotherapeutic treatment with exercises, *instruction*, *monitoring*, and *correction* are closely interwoven in the sense of an iterative adaptation [89]. This becomes evident to some extent in telerehabilitation with a direct connection to therapists. During a videoconference session, instruction, monitoring, and correction occur all at once. Even without direct videoconferencing, the therapist checks the training results via the aggregated information provided by the HET or via the recorded training video. This is then the basis upon which therapists give feedback for exercise correction, select a new exercise, or adjust the exercise instructions. This is done by changing the settings in games, creating and providing exercise videos, or by giving written feedback.

*Instructions* and *correction* via feedback from HETs through games are also frequently interwoven. Whenever a user is instructed with feedback to move within a certain range for success or failure, indirect *instruction* and *correction* via feedback are inseparable. This is similar to the procedure in physiotherapeutic processes with exercise treatment and adjustment, where the patient has to fulfill conditions with external attention focus. External focus leads to better motor skill learning than exercising with an internal attention focus [90]. The corresponding HETs have the potential to offer this procedure in the patient’s home environment at a high frequency, with many repetitions and with constant adaptation to the performance and ability of the patient. However, this interplay of *exercise instruction*, *monitoring*, and *correction* by HET is not described in detail. The adaptation of game tasks or game levels to simple motion parameters permits this conclusion. In the game “pluck the fruits,” for example, patients are instructed to achieve a certain ROM to pluck a fruit and advance to the next level. The HET indirectly corrects incorrect exercise execution by not allowing the fruit to be plucked, monitors exercise progress via ROM, and increases the ROM at the next level [42].

Exercise assistance solely from HETs is most often provided in the *instruction* of exercises, followed by *monitoring*, *assessment*, and *correction*. Simple, easily measurable, and presentable parameters such as *ROM*, *starting and final position* of the shoulder, and the *frequency* via the *number of repetitions* are by far the most frequently described parameters. Only rarely are parameters of movement quality used, such as *posture control*, *speed*, *harmony*, or *smoothness of movement*, which are also important for good exercise performance [91]. Beyond the pure ROM, it is important to avoid certain compensatory movements or to perform exercises with a smooth movement. This can serve to achieve a greater training effect, address certain muscle groups, or prevent negative consequences of the exercise. In addition, the quality of movement can be recorded in detail and reported back to the patient to improve exercise performance. This also makes it even more difficult to trick the system, for example, by replacing large movements with small fast movements.

Overall, some key components of motor learning are used for assistance by HETs but are not defined by the authors and developers. These are, for example, “observational learning” (eg, video-based instructions), “trial and error learning” (eg, in games with a task-oriented approach with feedback), and “errorless learning” (eg, the therapist adjusts the difficulty in the game via ROM) [92].

Only 3 HETs provide information on the relevance of exercises and changes in everyday life [20,58,70]. Even if it can be assumed that information and the motivation to exercise come from elsewhere, the integration of technology in these exercises to support and maintain motivation and adherence would be conceivable. It is therefore surprising that this aspect is not considered in many articles.

*Assessments* are usually represented by *ROM*. Very few technologies offer the possibility of patient-reported outcomes concerning *pain*, *strength*, and *function* [19,31,42]. *KiReS* offers an outstanding patient-specific approach in which therapists can create individually adapted questions [19]. Additional HETs with such functions would be desirable for patient-specific exercise therapy.

*Telerehabilitation aspects* are described in 27 of the 56 articles. This appears to be few and can be justified by the early development states. A total of 7 articles report *telerehabilitation aspects* as planned but not yet implemented. However, not all HETs seem to be designed for telerehabilitation, but rather for exercise assistance without connection to health care professionals. To what extent this is harmless and therapeutically useful is questionable as only 4 articles consider *adverse events*. Moreover, the vast majority of technologies do not offer a balanced exercise program for the shoulder. Balanced in this context is an exercise program that is adapted to the patient’s individual functional problem or is at least a complete exercise program that follows scientific training aspects. The individual adaptation of exercises to a patient is only partial and rarely done directly by HETs. In contrast to the physiotherapeutic treatment with repetitive adjustment [89], customization of exercises usually occurs at the beginning. The most common criterion for this is the *ROM*. More complex adjustments are only made in HETs with telerehabilitation. Usually, it is the responsibility of therapists who change the settings of an HET or teach patients to use the technology. This may protect patients at the current stage of HET development from physical damage as a result of incorrect exercising.

#### HET Components

Although some articles describe the *sensor hardware*, *hardware for output*, and *software* in detail, several do not. A lot of information is missing in the articles without detailed description, making traceability and comparability with other approaches impossible. For example, 28 of the 32 articles do not specify the version of the Kinect camera used. However, such information is important for drawing conclusions on the accuracy of joint position calculations [93,94].

The same applies to the lack of specification of the sensors used, whether they are body-worn sensors or sensors in the smartphone. This is also evident in some functions. Concerning the reminder function, for example, how it has been implemented remains open in most articles.

Microsoft’s Kinect depth-image camera seems to be particularly well suited to assist patients with musculoskeletal shoulder disorders in their exercises [95]. It was by far the most common sensor hardware, followed by IMUs and conventional color cameras. With regard to the detection accuracy of joint positions, the Kinect camera may be inferior to some marker-based, body-worn sensors. However, the Kinect’s advantage is a contactless measurement of the shoulder joint angle with acceptable accuracy, even though factors such as loosely fitting clothing can influence the accuracy [96].

Most the software is self-developed. This allows the adaptation of HETs to patients’ needs and becomes all the more apparent when more patients are involved in the development process. Development processes with or without user involvement are reported in varying degrees of detail. Rarely found was a reference to a strict development scheme (eg, a development according to the Medical Device Regulation [97]). This may be due to the current state of development. Nevertheless, development according to legal requirements and subsequent quality assurance for use in therapy would be advisable.

#### Development and Evaluation

Many technologies are not yet sufficiently developed. Instead, the focus is on the description and testing of technical components. Most of the articles are in “phase 0,” and only 5 articles report on *phase 3*. A systematic completion of all phases, comparable to drug and medical device studies with the resulting comparability and quality assurance, cannot be observed.

Interdisciplinary HETs focus more often on patient-relevant goals and correct exercises with the therapist in charge. Additional functions such as *reminders* or the *provision of information* were almost exclusively the result of interdisciplinary developments. It can be assumed that the patients’ or therapists’ experiences are responsible for this. *Interdisciplinary development* seems to be a reasonable approach to consider all relevant aspects and to develop sustainable practical solutions.

The results on *feasibility*, *acceptance*, and *usability* of the presented HETs are mainly positive. However, several articles report small sample sizes or tests with healthy persons. Therefore, it is often unclear to what extent these results are transferable to patients and practice.

Different measuring instruments and study designs are used. *Feasibility* is mostly tested under laboratory conditions. Using different study designs and measurement tools, 5 articles in *phase 3* and 3 articles in *phase 2* show significant improvements in at least one shoulder-relevant outcome parameter. In contrast, 6 articles reported insignificant results. For these studies, which had small sample sizes and were tested for superiority or without a control group, the technologies cannot automatically be considered unsuitable. Standardized comparable parameters would be necessary for meta-analyses in the presence of further randomized controlled trials.

### Limitations

The deliberately broad database search resulted in a high number of records. In several attempts to specify search terms, this led to a reduction in the number of records and a loss of relevant articles. As a consequence, the decision was made to screen a large number of records for this scoping review. Nevertheless, it is only a broad overview of the scientific literature. A supplementary market analysis of HETs that assist patients with musculoskeletal shoulder disorders in their exercises has not been conducted.

Following a pilot data extraction, 1 reviewer performed the content analysis of the full texts. A content analysis of all full texts by 2 reviewers separately may have led to more reliable results.

This scoping review serves exclusively as a broad overview of HETs that assist patients with musculoskeletal shoulder diseases in their home-based exercises. Quality assessments of the studies and a meta-analysis were not done amid the different study designs with predominantly small sample sizes. Therefore, this review does not provide systematically substantiated answers in this respect. The aim was to identify and analyze the development and use of HETs describing their approaches and their stage of development. A narrower limitation and subdivision, for example, according to development status or hardware use, would be useful as a next step. A deeper analysis and presentation within subgroups would be possible.

### Conclusions

This scoping review provides an overview of HETs that assist patients with musculoskeletal shoulder disorders in their exercises at home. The spectrum of identified HETs ranges from simple videoconferencing systems, exergames, and apps without telerehabilitation aspects to complex sensor-based technologies for telerehabilitation. HETs assist patients *directly* or *indirectly* (eg, with exercises hidden in a game). Various *sensor hardware*, *hardware for output*, and *software* are used for *instruction*, *correction*, or *monitoring* of exercises and *assessments*. The *Microsoft Kinect camera* and *ROM* are most frequently used and well proven. Other parameters of *movement quality* (eg, *posture control* or *smoothness*) are rarely used but are also important for good exercise performance and movement learning. Few articles describe a technology-based *exercise reminder* or the *provision of information* (eg, how to modify daily activities according to the shoulder condition or explain the importance of exercises).

Although some HETs offer patients a balanced exercise program, although usually not individually, most HETs fall short of doing this. The support of evidence-based exercises based on guidelines, recovery processes, or expected rehabilitation phases is missing here. Exercise *adaptation* to an individual patient is mostly done by therapists and rarely by HETs.

Most HETs are not yet sufficiently developed, but rather are in a prototype state. Few HETs achieved significant improvements in at least one shoulder-relevant outcome parameter. Various instruments and study designs are used to evaluate *acceptance*, *usability*, or *effectiveness or efficacy*, mostly in small samples. Interdisciplinary developed HETs more often define their target group, focus on patient-relevant goals, and offer additional functions such as reminders or extra information. Health care professionals and patients should therefore be involved in the product development cycle to consider all relevant aspects of sustainable practical HETs. This includes the embedding of an HET in the care process, prototype testing as well as usability and acceptance tests with the later target group under real-life conditions. A greater correspondence of study designs with control groups for effectiveness and efficacy studies, comparable standardized assessment instruments, and larger sample sizes would enable better comparability and, consequently, a sound selection of HETs for clinical use. Altogether, this review provides a first overview and thus a basis for pursuing more specific questions in the future about subgroups of HETs for selection or recommendation for clinical use as well as for further research and development.

## Appendix

Multimedia Appendix 1 Search queries.

Multimedia Appendix 2 Overview of results.

Multimedia Appendix 3 Overview of identified articles and related health-enabling technologies with telerehabilitation aspects.

## Abbreviations

HET: health-enabling technology
IMU: inertial measurement unit
KiReS: Kinect-based telerehabilitation system
PICO: Patient or Population, Intervention, Comparison, Outcome
PRISMA: Preferred Reporting Items for Systematic Reviews and Meta-analysis
RGB image: Red Green Blue image
ROM: range of motion
