# Supplementary material for: Health-Enabling Technologies to Assist Patients With Musculoskeletal Shoulder Disorders When Exercising at Home: Scoping Review
Source: JMIR Rehabil Assist Technol. 2021 Feb 4;8(1):e21107. doi: 10.2196/21107 (PMC8294637; doi:10.2196/21107)
Supplement: Multimedia Appendix 1 [file rehab_v8i1e21107_app1.docx]

**Search Queries**

**Medline via Pubmed**

| ((("upper limb"[Title/Abstract] OR shoulder[Title/Abstract] OR glenohumeral[Title/Abstract] OR "upper extremity"[Title/Abstract] OR "upper extremity"[MeSH Terms])  AND  (rehabilitation[Title/Abstract] OR rehabilitation[MeSH Terms] OR training[Title/Abstract] OR exercise*[Title/Abstract] OR "physical therapy modalities"[MeSH Terms] OR exercise[MeSH Terms] OR "physical therapy"[Title/Abstract] OR physiotherapy[Title/Abstract] OR "computer-assisted therapy"[Title/Abstract] OR "therapy, computer-assisted"[MeSH Terms] OR "technology-assisted therapy"[Title/Abstract]) AND (virtual[Title/Abstract] OR game*[Title/Abstract] OR "video games"[MeSH Terms] OR exergame*[Title/Abstract] OR gami*[Title/Abstract] OR tele[Title/Abstract] OR telerehabilitation[Title/Abstract] OR tele-rehabilitation[Title/Abstract] OR telerehabilitation[MeSH Terms] OR digital[Title/Abstract] OR automated[Title/Abstract] OR technological[Title/Abstract] OR technology[Title/Abstract] OR technology[MeSH Terms] OR technical[Title/Abstract] OR "computer system"[Title/Abstract] OR "computer systems"[Title/Abstract] OR "computer systems"[MeSH Terms] OR hardware[Title/Abstract]) |
| --- |

**EMBASE via Ovid**

| *('upper limb':ab,ti OR 'upper limb'/de OR shoulder:ab,ti OR 'shoulder'/de OR glenohumeral:ab,ti OR 'upper extremity':ab,ti OR 'arm'/de) AND (rehabilitation:ab,ti OR 'rehabilitation'/de OR training:ab,ti OR 'training'/de OR exercise*:ab,ti OR 'exercise'/de OR 'physical therapy':ab,ti OR physiotherapy:ab,ti OR 'physiotherapy'/de OR 'computer-assisted therapy':ab,ti OR 'computer-assisted therapy'/de OR 'technology-assisted therapy':ab,ti)*  *AND*  *('virtual':ab,ti OR 'game*':ab,ti OR 'gami*':ab,ti OR 'exergame*':ab,ti OR 'tele*':ab,ti OR 'telerehabilitation'/de OR 'telerehabilitation':ab,ti OR 'digital':ab,ti OR 'automated':ab,ti OR 'technological':ab,ti OR 'technology'/de OR 'technology':ab,ti OR 'technical':ab,ti OR 'computer system'/de OR 'computer system' OR 'hardware':ab,ti)*  *AND*  *([article]/lim OR [conference paper]/lim OR [review]/lim OR [short survey]/lim) AND [humans]/lim AND [1997-2019]/py* |
| --- |

**IEEE**

| ("upper limb" OR shoulder OR glenohumeral OR "upper extremity" OR "Mesh_Terms":"upper extremity") AND (rehabilitation OR "Mesh_Terms":rehabilitation OR training OR excercise* OR "Mesh_Terms":"physical therapy modalities" OR "Mesh_Terms":excercise OR "physical therapy" OR physiotherapy OR "computer-assisted therapy" OR "Mesh_Terms":"therapy, computer-assisted" OR "technology-assisted therapy")  AND  (virtual OR game*OR "Mesh_Terms":"video games" OR exergame* OR gami* OR tele OR telerehabilitation OR tele-rehabilitation OR "Mesh_Terms":telerehabilitation OR digital OR automated OR technological OR technology OR "Mesh_Terms":technology OR technical OR "computer system" OR "computer systems" OR "Mesh_Terms":"computer systems" OR hardware) |
| --- |

**CINAHL**

| *(TI "upper limb" OR AB "upper limb" OR MJ "upper limb" OR TI shoulder OR AB shoulder OR MJ shoulder OR TI glenohumeral OR AB glenohumeral OR MJ glenohumeral OR TI "upper extremity" OR AB "upper extremity" OR MJ "upper extremity" OR MJ arm) AND (TI rehabilitation OR AB rehabilitation OR MJ rehabilitation OR TI training OR AB training OR MJ training OR TI exercise* OR AB exercise* OR MJ exercise OR TI "physical therapy" OR AB "physical therapy" OR TI physiotherapy OR AB physiotherapy OR MJ physiotherapy OR TI "computer-assisted therapy" OR AB "computer-assisted therapy" OR MJ " therapy, computer-assisted " OR TI "technology-assisted therapy" OR AB "technology-assisted therapy")*  *AND*  *(TI virtual OR AB virtual OR TI game* OR AB game* OR MJ "video games" OR TI exergame OR AB exergame OR TI gami* OR AB gami* OR TI tele OR AB tele OR TI telerehabilitation OR AB telerehabilitation OR MJ telerehabilitation OR TI tele-rehabilitation OR AB tele-rehabilitation OR TI digital OR AB digital OR TI automated OR AB automated OR TI technological OR AB technological OR TI technology OR AB technology OR MJ technology OR TI technical OR AB technical OR TI "computer system" OR AB "computer system" OR TI "computer systems" OR AB "computer systems" OR MJ "computer systems" OR TI hardware OR AB hardware)* |
| --- |

**PEDro**

| 32 Single search queries in abstract and title with settings Body Part: upper arm, shoulder or shoulder girdle; Published since: 1997;  First search query: Subdiscipline: musculoskeletal  Second search query: Subdiscipline: Orthopaedic  Search terms:  Virtual  game*  “video games”  exergame*  gami*  tele* Subdiscipline:  telerehabilitation  tele-rehabilitation  digital  automated  technological  technology  technical  **"**computer systems**"**  **"**computer system**"**  Hardware |
| --- |

**Scopus**

| ( TITLE-ABS-KEY ( "upper limb" ) OR TITLE-ABS-KEY ( shoulder ) OR TITLE-ABS-KEY ( glenohumeral ) OR TITLE-ABS-KEY ( "upper extremity" ) OR TITLE ( arm ) ) AND ( TITLE-ABS-KEY ( *rehabilitation ) OR TITLE-ABS-KEY ( training ) OR TITLE-ABS-KEY ( exercise* ) OR TITLE-ABS-KEY ( "physical therapy" ) OR TITLE-ABS-KEY ( physiotherapy ) OR TITLE-ABS-KEY ( "computer-assisted therapy" ) OR TITLE-ABS-KEY ( "technology-assisted therapy" ) )  AND  ( TITLE-ABS-KEY ( virtual ) OR TITLE-ABS-KEY ( *game* ) OR TITLE-ABS-KEY ( "video games" ) OR TITLE-ABS-KEY ( *gami* ) OR TITLE-ABS-KEY ( tele ) OR TITLE-ABS-KEY ( telerehabilitation ) OR TITLE-ABS-KEY ( tele-rehabilitation ) OR TITLE-ABS-KEY ( digital ) OR TITLE-ABS-KEY ( automated ) OR TITLE-ABS-KEY ( technological ) OR TITLE-ABS-KEY ( technology ) OR TITLE-ABS-KEY ( technical ) OR TITLE-ABS-KEY ( "computer system*" ) OR TITLE-ABS-KEY ( hardware ) )  AND  PUBYEAR > 1996 AND ( LIMIT-TO ( DOCTYPE , "ar" ) OR LIMIT-TO ( DOCTYPE , "cp" ) OR LIMIT-TO ( DOCTYPE , "re" ) OR LIMIT-TO ( DOCTYPE , "sh" ) ) |
| --- |
