# Supplementary material for: Health-Enabling Technologies to Assist Patients With Musculoskeletal Shoulder Disorders When Exercising at Home: Scoping Review
Source: JMIR Rehabil Assist Technol. 2021 Feb 4;8(1):e21107. doi: 10.2196/21107 (PMC8294637; doi:10.2196/21107)
Supplement: Multimedia Appendix 3 [file rehab_v8i1e21107_app3.pdf]

Overview of identified articles and related health-enabling technologies with telerehabilitation aspects

	Name of health-enabling technology	Study	Details
**Telerehabilitation**			
	Kinect-based telerehabilitation system	Anton et al [18,19]	App; telerehabilitation with Kinect-based exercise guidance and therapist decision support
	Unstated	Macias-Hernandez et al [20]	App; telerehabilitation with video recording and written feedback
	iJoint App	Ongvisatepaiboon et al [21-23]	App; telerehabilitation with smartphone IMU[a]
	Unstated	Pastora-Bernal et al [24,25]	App; telerehabilitation with web-based exercise program (shows video, images, and parameters)
	TeRa	Cabana et al [26]; Tousignant et al [27]	Telerehabilitation with biomedical sensors
	Unstated	Eriksson et al [28,29]	Telerehabilitation with a videoconferencing system
	Unstated	Budziszewski [30]	Game; app; telerehabilitation with head-mounted display, body-wearable controller (Razer Hydra), and videoconferencing system
	SHOULPHY app	Carbonaro et al [31]; Lucchesi et al [32]	Game; app; telerehabilitation with IMUs
	Unstated	Chang et al [33,34]	Game; app; telerehabilitation with shoulder wheel with control module

	iJoint	Chiensriwimol et al [35,36]	Game; app; telerehabilitation with smartphone IMUs and audio-biofeedback module
	MoMo	Chung and Chen [37]	Game; app; telerehabilitation with wearable devices not specified
	InMotion	Pinto et al [38]	Game; app; telerehabilitation with Kinect-based exercise guidance
	Unstated	Postolache et al [39]	Game; app; telerehabilitation with Kinect-based exercise guidance
	Unstated	Postolache et al [40]	Game; app; telerehabilitation with Kinect-based exercise guidance
	GEAR	Rahman et al [41,42]	Game; app; telerehabilitation with IMUs
	PARC	Symeonidis and Kavallieratou [43]	Game; app; telerehabilitation with Kinect-based exercise guidance
	Unstated	Viegas et al [44]	Game; app; telerehabilitation with Kinect- and IMU-based exercise guidance
	Cloud motion-sensing rehabilitation system	Yeh et al [45]	Game; app; telerehabilitation with Kinect- and IMU-based exercise guidance
	Unstated	Ying and Aimin [46]	Game; app; telerehabilitation with marker–based augmented reality exercise guidance

**Telerehabilitation planned**			
	Unstated	Huang et al [47]	Game; app
	Unstated	Mangal et al [48]	Game; app
	Unstated	Yeh et al [49]	Game; app
	BANDCIZER (TM)	McGirr et al [50]	Monitoring only
	GoNet v2	Neto et al [51]	Monitoring only
	Unstated	Uttarwar and Mishra [52]	Monitoring only
	I-FLEXBAR	Shieh et al [53]	Flexbar pressure sensor–based exercise feedback; telerehabilitation planned
**No telerehabilitation**			
	Unstated	Choi et al [54]	App; smartphone-based exercise guidance and IMU-based angle feedback
	Shoulder physiotherapy application	Cubukcu and Yuzgec [55]	App; video-based exercise instruction; Kinect-based feedback
	Unstated	Dahl-Popolizio et al [56]	App; playful tasks
	ZOUZI	Du et al [57]	App; IMU-based exercise feedback
	Unstated	Stütz et al [58]	App; video-based exercise instruction; IMU-based range of motion measurement
	Zishi	Wang et al [59]	App; IMU-, smart garment–, and modular soft sensor–based exercise guidance
	Unstated	Quevedo et al [60]	App; playful tasks
	Unstated	Chen [61]	Playful tasks with virtual (Kinect-based) finger ladder and single curved shoulder

	Unstated	Arif et al [62]	Game; app
	2012 unstated; 2016 MirrARbilitation	Da Gama et al [63-65]	Game; app; Kinect–based exercise guidance
	Unstated	Du et al [66]	Game; app
	Unstated	Fernandez-Cervantes et al [67]	Game; app
	The Sorcerer's Apprentice	Fikar et al [68]	Game; app
	KineActiv R	Muñoz et al [69]	Game; app
	Unstated	Nava et al [70]	Game; app
	Commercial (Wii Sports)	Pekyavas and Ergun [71]	Game; app
	Unstated	Powell and Powell [72]	Game; app
	Commercial (Wii sports)	Rizzo et al [73]	Game; app
	Unstated	Shi and Peng [74]	Game; app
	Commercial Xbox 360 Kinect	Wiederhold and Wiederhold [75]	Game; app
	Unstated	Yin and Xu [76]	Game; app
	Commercial Xbox 360 Kinect	Arman et al [77]	Game
	Rehabilitation gaming system	Chen et al [78]	Game
	Unstated	Gorsic and Novak [79]; Gorsic et al [80]	Game
	Unstated	Gutiérrez et al [81]	Game
	Unstated	Kanbe et al [82]	Game
	Commercial interactive rehabilitation exercise system	Sveistrup et al [83]	Game
	Unstated	Ar and Akgul [84]	Monitoring only
	Unstated	Chen et al [85]	Monitoring only
	Unstated	Chiang et al [86]	Monitoring only
	Unstated	Tekriwal and Pandian [87]	Monitoring only

[a]IMU: inertial measurement unit
